# Early Mortality in Infants Born with Neonatally Operated Congenital Heart Defects and Low or Very-Low Birthweight: A Systematic Review and Meta-Analysis

**DOI:** 10.3390/jcdd10020047

**Published:** 2023-01-27

**Authors:** Neil Derridj, Ali Ghanchi, Damien Bonnet, Pauline Adnot, Makan Rahshenas, Laurent J. Salomon, Jérémie F. Cohen, Babak Khoshnood

**Affiliations:** 1 Centre of Research in Epidemiology and Statistics (Inserm 1153, CRESS), Université Paris Cité, 75004 Paris, France; 2 M3C-Necker, National Reference Center for Complex Congenital Heart Diseases, APHP, Université Paris Cité, Hôpital Necker-Enfants Malades, CEDEX 15, 75743 Paris, France; 3 Department of Obstetrics and Fetal Medicine, APHP, Université Paris Cité, Hôpital Necker-Enfants Malades, CEDEX 15, 75743 Paris, France; 4 Department of General Pediatrics and Pediatric Infectious Diseases, APHP, Université Paris Cité, Hôpital Necker-Enfants Malades, CEDEX 15, 75743 Paris, France

**Keywords:** congenital heart defects, systematic review, meta-analysis, low birth weight, hypoplastic left heart syndrome, mortality, coarctation of the aorta, transposition of the great arteries, total anomalous pulmonary venous return

## Abstract

Mortality outcomes of children with isolated neonatally operated congenital heart defects (CHDs) born with a low (LBW), moderately low (MLBW) or very-low birthweight (VLBW) remain ambiguous. We searched Medline and Embase (inception until October 2021) and included studies that evaluated early mortality. The risk of bias was assessed using the Critical Appraisal Skills Program cohort checklist. Meta-analysis involved random-effects models. We explored variability in mortality across birthweight subgroups, CHD types, and study designs. From 2035 reports, we included 23 studies in qualitative synthesis, and the meta-analysis included 11 studies (1658 CHD cases), divided into 30 subcohorts. The risk of bias was low in 4/11 studies included in the meta-analysis. Summary mortality before discharge or within one month after surgery was 37% (95%CI 27–47). Early mortality varied by birthweight (VLBW 56%, MLBW 15%, LBW 16%; *p* = 0.003) and CHD types (hypoplastic left heart syndrome (HLHS) 50%, total anomalous pulmonary venous return (TAPVR) 47%, transposition of the great arteries (TGA) 34%, coarctation of the aorta (CoA) 16%; *p* = 0.13). Mortality was higher in population-based studies (49% vs. 10%; *p* = 0.006). One-third of infants born with neonatally operated isolated CHDs and LBW, MLBW, or VLBW died within 30 days after surgery. Mortality varied across infant and study characteristics. These results may help clinicians assess neonatal prognosis. PROSPERO registration CRD42020170289.

## 1. Introduction

The reported prevalence of congenital heart defects (CHDs) worldwide continues to increase and is currently estimated in 9.5/1000 live births [1]. Up to 8 to 15% of children with CHDs are also born with a low birthweight [2,3,4] (LBW; birthweight < 2500 g) or very-low birthweight (VLBW; birthweight < 1500 g) [5,6]. Although the prevalence of children born with an LBW varies according to the specific CHD subtype [7,8], this association can potentially affect prognosis. This is especially true for neonatally operated CHDs (OCHDs) for which surgery cannot be delayed.

Numerous studies focusing on mortality report data on children born with a LBW. However, these reports are heterogeneous in design, include a small number of patients, are mainly tertiary centers studies, or often involve only limited subtypes of OCHDs [8,9]. Furthermore, the CHDs included in these studies are not sorted between isolated CHDs and CHDs associated with other congenital anomalies and/or genetic syndromes. It is of note that CHDs associated with multiple malformations/syndromes are a major confounding factor for morbidity and mortality outcomes. For these reasons, the direct effect of LBW associated with the OCHD or OCHD subtypes on mortality is not clearly established. The potential increase in death related to the combined effect of LBW and OCHD remains insufficiently described and ambiguous. This information is yet essential to advise parents on the predicted outcomes when their child is born with a LBW and will undergo early cardiac intervention. Here, we sought to provide a comprehensive qualitative and quantitative synthesis of early mortality and morbidity for children born with a LBW and a CHD, and to investigate the potential sources of variability in outcomes across studies and subgroups.

## 2. Materials and Methods

This study is reported in accordance with Preferred Reporting Items for Systematic Review and Meta-analyses (PRISMA) guidelines (Supplementary Materials: Table S1) [10]. The review protocol was registered in PROSPERO (CRD4202170289) [11]. Discrepancies between the protocol and the final review are reported in the Supplementary Materials: Table S2.

### 2.1. Eligibility Criteria

We included prospective and retrospective cohort studies investigating early morbidity and mortality outcomes in infants with OCHD born with a LBW or VLBW. CHDs were defined as children born with a structural heart defect and excluded patent ductus arteriosus, cardiac tumors, cardiomyopathies, and arrhythmias. Isolated CHDs were defined as CHDs not associated with chromosomal anomalies, malformations from other systems, or syndromes [12].

When accuracy about the isolated CHD status was not specified, articles on hypoplastic left heart syndrome (HLHS), aortic stenosis (AS), Ebstein’s anomaly, total anomalous pulmonary venous return (TAPVR), transposition of the great arteries (TGA) and coarctation of the aorta (CoA) were, however, retained [13], as these specific defects are rarely associated with extracardiac anomalies and/or genetic syndromes. OCHDs included all isolated CHDs operated on in the neonatal period, defined as the time elapsed between birth and 28 days of age.

We used the World Health Organization’s definition of LBW (i.e., birthweight < 2500 g) and VLBW (i.e., birthweight < 1500 g) [14]. A moderately low birthweight (MLBW) referred to infants with a birthweight between 1500 g and <2499 g.

Early morbidity and mortality outcomes during infancy were analyzed. Morbidity was defined as a state of ill health resulting from a disease or condition (symptomatic or sequela) [15]. Long- and short-term morbidity indicators included necrotizing enterocolitis (NEC), bronchopulmonary dysplasia (BPD), neurodevelopmental outcomes, and other outcomes depending on data availability. Early mortality was defined as proportionate mortality before discharge or within one month after surgery [16], expressed as a percentage.

Conference abstracts, case reports, CHDs and single umbilical artery, outcomes in the offspring of women born with CHDs, and studies with missing data for birthweight outcomes were excluded.

### 2.2. Literature Search Strategy and Study Selection

A comprehensive literature search was carried out on Medline (via Pubmed) and Embase databases with the assistance of an information specialist.

Medical Subject Headings (MeSH)/Medical Embase Medical Headings (EMTREE) and free text keywords included different synonyms for CHD, CHD subtypes, LBW, and VLBW, and were combined using Boolean operators, as described in the Supplementary Materials: Table S3.

The search was conducted from inception onwards, and no language restrictions were applied. A manual search of references in the included articles was also carried out.

First, titles and abstracts of the retrieved studies were screened independently by two reviewers (N.D. and A.G.) and using the Rayyan web application [17]. Then, they retrieved the full texts of the relevant articles and evaluated them for inclusion.

### 2.3. Data Extraction

A predetermined data extraction form was designed and used independently by two reviewers (N.D. and A.G.). Extracted data for each study included study characteristics, type/subgroup of OCHD, study population (i.e., LBW, MLBW and VLBW), outcomes, data sources, study design, inclusion and exclusion criteria. Study authors were contacted to request further information or clarification of results when needed. In the case of multiple reports of the same cohort, only the most extensive report in terms of sample size and the latest publication was included.

### 2.4. Risk of Bias Assessment

We assessed the risk of bias using the Critical Appraisal Skills Program (CASP) cohort study checklist [18]. The checklist contains 12 questions that are divided into three sections that enable a structured approach to finding evidence, determining possible sources of bias, and evaluating the internal and external validity of each study. We adapted this checklist to our study question paying particular attention to selection, confounding, and measurement biases.

Throughout the entire review process (article selection, data extraction, and risk of bias assessment), discrepancies were resolved through discussion. Any further disagreements between the two reviewers (N.D. and A.G.) were resolved by a third reviewer (B.K.).

### 2.5. Data Synthesis and Statistical Analysis

Certain studies were subdivided for the purpose of the review. Data regarding each relevant OCHD subtype were subdivided to obtain one cohort per OCHD subtype. Each subcohort was further stratified by birthweight (LBW, MLBW, and VLBW). For example, if a study included LBW and VLBW infants with TGA and CoA, the study was subdivided into four subcohorts, each capturing one type of CHD and one group of birthweight (e.g., TGA and LBW). When the data allowed, we extracted outcomes separately for MLBW and VLBW.

A meta-analysis of proportions (with their 95% confidence intervals) was carried out using random-effects models with inverse variance weighting, using the Simonian and Laird method [19,20]. Heterogeneity was assessed using the *I*^2^ statistic and visual inspection of forest plots. From the data available in the studies, we first estimated the overall summary mortality across all studies, birthweight categories, and four OCHD subtypes (we were unable to obtain data for AS and Ebstein’ anomaly). Then, analyses were stratified by the type of OCHD (HLHS, TAPVR, TGA, CoA), birthweight (LBW, MLBW, VLBW), and study design (population-based vs. hospital-based). A meta-regression was performed to assess the effect of these study-level variables, using a logit-normal random-effects model; meta-regression coefficients were exponentiated to obtain the odds ratios of mortality.

Publication bias was evaluated through visual inspection of the funnel plot and by Egger’s test. Sensitivity analysis was performed by repeating the main meta-analysis on studies judged at a low overall risk of bias. Statistical analysis involved the use of the *metan*, *metaprop*, *metareg*, and *metabias* commands in Stata/SE version 15 (Stata Corp, College Station, TX, USA); *p* values < 0.05 were considered statistically significant.

## 3. Results

### 3.1. Literature Searches

**Figure 1:**
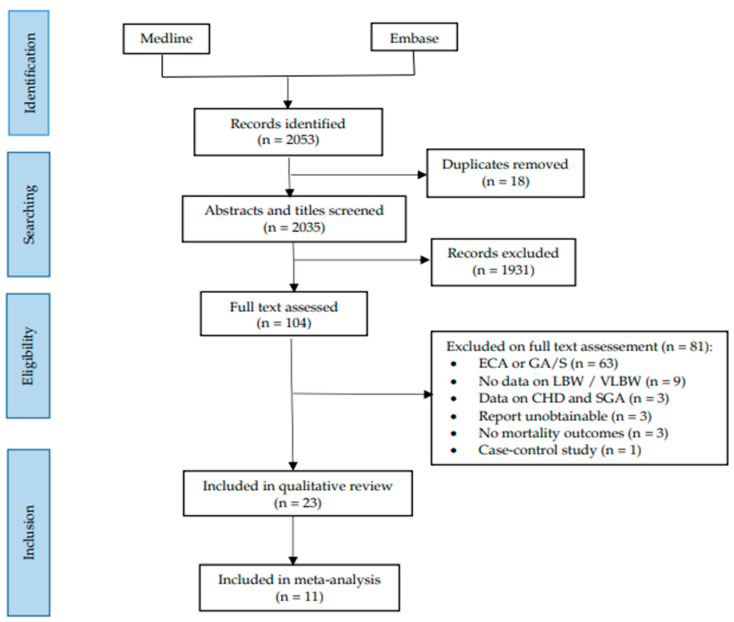
PRISMA flowchart. Legend: ECA, extracardiac anomaly; GA/S, genetic anomaly and/or syndrome; LBW, low birthweight; SGA, small for gestational age; VLBW, very-low birthweight.

The search conducted on 13 October 2021, identified 2053 potentially relevant publications, of which 104 articles were assessed for eligibility. No additional studies were found from hand-searching reference lists. In total, 23 articles were included in this review, of which 11 studies contained sufficient data for a meta-analysis (Figure 1).

### 3.2. Characteristics of Included Studies

Characteristics of the included studies are presented in Table 1. Overall, the majority of studies were published after the year 2011 and were from the US (74%). The duration of studies ranged between 1 year [21] and 26 years [22,23]. The total number of infants born with CHDs included in the studies ranged between 25 [24] and 98,523 [25]. More than half (62%) of the included studies were population-based studies.

The type of CHD associated with an LBW and/or VLBW varied in the included studies. There were two publications (9%) that included all isolated CHDs [25,26]. Most studies were on HLHS (74%), although several authors studied multiple specific CHDs concomitantly. Less than half of the studies (40%) evaluated the adverse outcomes in VLBW infants, although more than a quarter (26%) used multiple populations (e.g., VLBW and LBW) in the same study.

Although multiple outcomes were studied simultaneously, infant mortality was the main reported outcome across the studies (21 studies, 91%). Heterogeneous mortality indicators were used in the studies, with most authors reporting mortality following surgical intervention (18 studies, 78%). Proportionate mortality was used in 16 studies (70%), and death before discharge or 30 days after surgery was the most frequent mortality outcome (11 studies, 48%).

Morbidity indicators included a range of heterogeneous outcomes, such as duration of respiratory support, seizures, sepsis, and hypoglycemia. NEC was the main morbidity indicator, assessed in a third of the studies (30%; Supplementary Materials: Table S4).

**Table 1:** Characteristics of the studies included in the qualitative review (*n* = 23).

Study	Year	Duration	Location	N CHD	All iCHD	Other Specific CHD	Population	Outcome(s)
Anderson [27] ^§ +^	2014	1997–2012	USA	299	No	HLHS, AS, Ebs, TAPVR and CoA	VLBW	Discharge mortality
Archer [21] ^§ +^	2011	2006–2007	USA	893	No	HLHS, AS, TAPVR, TGA and CoA	VLBW	Mortality at discharge or 1 year
Bacha [28] ^+^	2001	1990–1999	USA	189	No	CoA	VLBW	Hospital and 5-year survival
Bain [29] ^§ +^	2014	1997–2010	USA	98,523	No	SD	VLBW	NEC
Best [26] ^§^	2017	1985–2003	UK	5093	Yes	AS, TGA and CoA	LBW	Overall survival and 5-year survival for cohort born in 2003
Curzon [3] ^§^	2008	2002–2004	USA	3022	No	HLHS, TAPVR, TGA and CoA	LBW	90-day post-op
El Hassan [30] ^§^	2018	2004–2013	USA	5720	No	HLHS	SGA and LBW	Hospitalization mortality and NEC
Fisher [31] ^§ +^	2015	2006–2011	USA	1931	No	HLHS, AS, TAPVR, TGA and CoA	VLBW	NEC
Gelehrter [32] ^+^	2011	1998–2007	USA	47	No	HLHS	SGA and LBW	Transplant-free survival through Fontan palliation
Hirsch [33] ^§^	2011	1992–2005	USA	406	No	HLHS	LBW	Survival and mortality at 1 year after surgical intervention
Kalfa [34]	2014	2006–2012	USA	146	No	HLHS, Ebs, TAPVR, TGA and CoA	LBW	Mortality before discharge or within 30-days post-op and immediate post-operative outcomes (ECMO, NEC, length of mechanical ventilation, delayed chest closure, prolonged inotropic support, renal failure, and cardiac arrest)
Kalfa [9]	2015	2006–2014	USA	28	No	HLHS	LBW	Death before discharge or 30-days post-op, delayed chest closure, cardiac arrest, length of mechanical ventilation, arrhythmia, pulmonary complications, renal failure, and NEC
Karamlou [35]	2009	1993–2004	USA	36	No	CoA	LBW	1-year survival
Manchego [36]	2018	2003–2016	Australia	171	No	HLHS, TGA and CoA	LBW	Survival and post-operative complications (unplanned re-intervention, ECMO, sepsis, stroke, NEC, etc., for all CHDs only) at 6 months,
Miller [37] ^§^	2019	2005–2008	USA	509	No	HLHS	SGA and LBW	6-year mortality, neurodevelopment, hospital length of stay, unplanned re-intervention and quality of life
Murphy [38]	2015	2005–2013	UK	41	No	HLHS	LBW	Midterm survival after initial hybrid procedure
Oh [39] ^§^	2017	1992–2014	New Zealand	133	No	HLHS	LBW	Mortality before discharge or within 30-days post-op
Oppido [40]	2004	1993–2002	Italy	60	No	HLHS, TGA and CoA	LBW	Inpatient mortality (within 30 days after operation)
Oster [22] ^§^	2013	1979–2005	USA	415	No	CCHD	LBW	1-year survival
Pappas [25] ^§ +^	2012	1998–2005	USA	110	Yes	HLHS, TAPVR, TGA and CoA	VLBW	Discharge mortality and in hospital morbidity (seizures, NEC, ICH, PVL, BPD, neurological impairment, premature retinopathy, Apgar score, late-onset sepsis)
Roussin [24] ^+^	2007	1990–2003	France	25	No	TGA	LBW, SGA and VLBW	Discharge mortality and early morbidity (prolonged inotropic support, cardiac ischemia, pulmonary hypertension, prolonged ventilation, neurologic disease)
Shepard [41] ^§^	2010	1982–2006	USA	450	No	HLHS, TAPVR and CoA	MLBW and VLBW	30-day survival
Siffle [23] ^§^	2015	1979–2005	USA	212	No	HLHS	LBW and VLBW	Overall survival between 1979–2005

Legend: ^§^ population-based study; ^+^ preterm births only. BPD, bronchopulmonary dysplasia; ECMO, extracorporeal membrane oxygenation; iCHD, isolated CHD; CCHD, critical CHD; CSE, conditional survival estimate; ICH, intracranial hemorrhage; LBW, low birthweight; N CHD, total number of congenital heart defects cases; NEC, necrotizing enterocolitis; PVL, periventricular leukomalacia; SGA, small for gestational age; SD, septal defects; VLBW, very-low birthweight; HLHS, hypoplastic left heart syndrome; TAPVR, total anomalous pulmonary venous return; CoA, coarctation of the aorta; TGA, transposition of the great arteries; MLBW, moderately low birthweight.

### 3.3. Risk of Bias

The overall risk of bias across the studies is summarized in Table 2. All studies addressed a clearly focused issue and had sufficient follow-up of the cohorts. However, the quality of studies regarding other criteria in the checklist varied greatly. In particular, most studies were considered at risk of selection and measurement biases, especially with regard to diagnosis of CHDs using a validated diagnostic method. Four studies were deemed to have a high risk of selection bias [9,24,28,40]. Two thirds of the studies (15 studies, 65%) accounted for confounding factors (e.g., parity, ethnicity, maternal disease, socio-economic status). Consequently, the accuracy of the reported results also varied across the studies. Only seven studies (30%) showed detailed and precise results with relatively narrow confidence intervals which were comparable to one another. Notwithstanding differences in geographic locations, the external validity criterion was met for most studies (13 studies, 62%) as they were population-based and had large sample sizes.

Overall, out of the 23 studies included in this review we observed that 11 studies had a low risk of bias (i.e., overall risk of bias score >5, Table 2).

**Table 2:** Summary of the risk of bias assessment across the studies included in the qualitative review (*n* = 23).

Study	CASP Criteria							Total Score/9 ^ǂ^	Overall Risk of Bias **
	Focused Issue	Selection Bias §	Measurement Bias	Confounding	Follow-Up	Results ^§^	External Validity		
Anderson * [27]	+	+	-	-	+	-	+	4	High
Archer * [21]	+	+	-	-	+	++	+	6	Low
Bacha * [28]	+	-	-	+	+	-	-	3	High
Bain [29]	+	+	-	+	+	+	+	6	Low
Best [26]	+	+	+	+	+	+	+	7	Low
Curzon [3]	+	-	-	-	+	+	+	4	High
ElHassan * [30]	+	-	-	-	+	+	+	4	High
Fisher [31]	+	+	-	+	+	++	+	7	Low
Gelehrter [32]	+	-	-	+	+	+	-	4	High
Hirsch [33]	+	-	-	+	+	++	+	6	Low
Kalfa (2015) * [9]	+	+	-	-	+	-	-	3	High
Kalfa (2014) * [34]	+	+	+	+	+	+	-	6	Low
Karamlou [35]	+	++	-	-	+	-	-	4	High
Manchego [36]	+	+	+	-	+	-	-	4	High
Miller [37]	+	-	-	+	+	++	+	6	Low
Murphy [38]	+	++	+	-	+	-	-	5	High
Oh * [39]	+	-	+	+	+	+	+	6	Low
Oppido * [40]	+	-	+	-	+	-	-	3	High
Oster [22]	+	-	-	+	+	++	+	6	Low
Pappas * [25]	+	-	+	+	+	-	+	5	High
Roussin * [24]	+	-	+	-	+	-	-	3	High
Shepard * [41]	+	++	+	-	+	-	+	6	Low
Siffle [23]	+	-	-	+	+	++	+	6	Low

Legend: CASP, Critical Appraisal Skills Program; ++ strongly fulfilled criteria; + fulfilled criteria; - weakly fulfilled criteria. * included in meta-analysis on early mortality. ** A high risk of bias if score ≤ 5. ^§^ scored/2. ^ǂ^ total number of + (scored out of 9).

### 3.4. Early Mortality in Infants Born with OCHD

**Figure 2:**
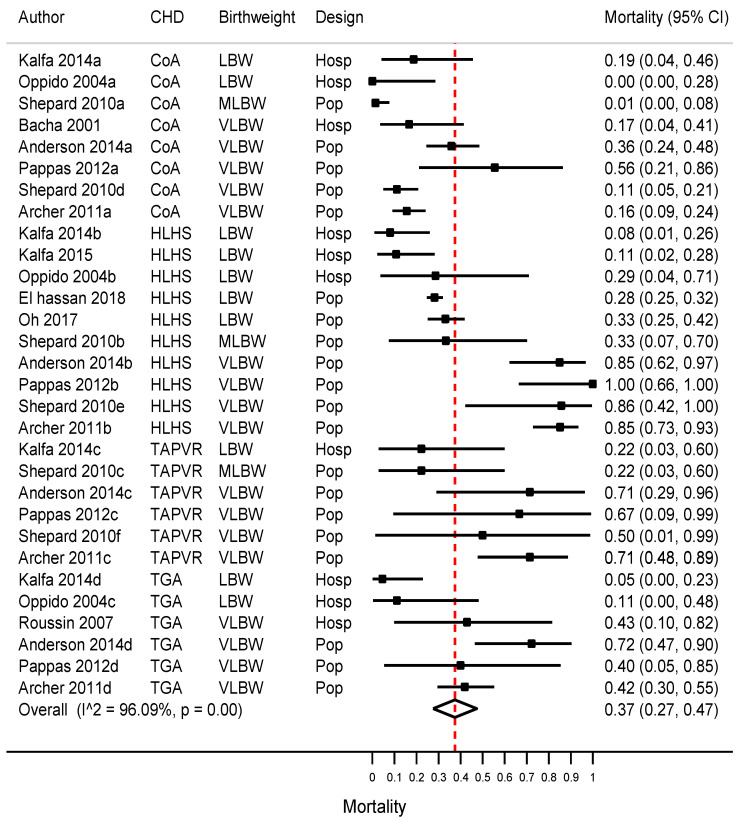
Early mortality in infants born with severe neonatally operated congenital heart defects and a low or very-low birthweight: Meta-analysis. Legend: CHD, congenital heart defect; LBW, low birthweight (i.e., <2500 g); MLBW, moderately low birthweight (i.e., 1500–2499 g), VLBW, very-low birthweight (i.e., <1500 g); HLHS, hypoplastic left heart syndrome; TAPVR, total anomalous pulmonary venous return; CoA, coarctation of the aorta; TGA, transposition of the great arteries; Hosp, hospital-based; Pop, population-based.

The main meta-analysis of mortality included data from 11 studies (subdivided into 30 cohorts) and encompassed 1445 CHD cases. The overall summary mortality before discharge or within one month after surgery was 37% (95%CI 27–47%, *I*^2^ 96%; Figure 2). Mortality varied across the CHD subtypes, but the difference was not statistically significant. Mortality for HLHS was 50% (30–69%, *I*^2^ 97%), TAPVR was 47% (26–72%, *I*^2^ 66%), TGA was 34% (11–58%, *I*^2^ 90%), and CoA was 16% (6–25%, *I*^2^ 88%) (*p* = 0.13; Table 3).

Mortality also varied according to the birthweight category: VLBW 56% (38–73%, *I*^2^ 95%), MLBW 15% (0–36%, *I*^2^ 68%) and LBW 16% (6–25%, *I*^2^ 93%) (*p* = 0.003; Table 3). Early mortality was much higher in population-based studies than in hospital-based studies: 49% (36–63%, *I*^2^ 97%) vs. 10% (4–15%, *I*^2^ 0%), respectively; *p* = 0.006.

### 3.5. Morbidity in Infants Born with an LBW or VLBW with a CHD

Comparable morbidity outcomes are summarized in the Supplementary Materials: Table S4. Morbidity indicators could not be pooled because of the insufficient number of studies with the same outcomes, heterogeneous definitions, and different measurements. The two main morbidity outcomes reported were NEC and neurodevelopmental anomalies. For NEC, Bain et al. reported increased risk of NEC for preterm VLBW infants with an atrial septal defect or ventricular septal defect [29], whereas this effect was not found in a population-based study that included all types of isolated CHDs [25]. Two studies reported neurodevelopmental disorders: one in toddlers born with a VLBW with an isolated CHD compared to VLBW infants without CHDs and one in children with HLHS born with an LBW vs. not LBW [24,37].

### 3.6. Additional Analyses

**Figure 3:**
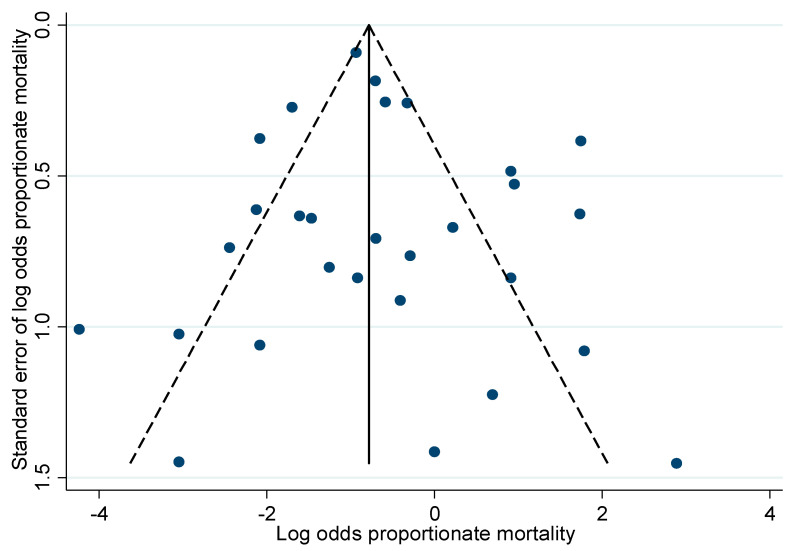
Funnel plot with pseudo 95% confidence limits.

Visual inspection of the funnel plot appeared symmetrical (Figure 3). Egger’s test did not show any statistical evidence for publication bias (*p* = 0.48). Following risk of bias assessment, 15 study cohorts (50%) were judged at a low overall risk of bias. Sensitivity analysis showed that early mortality was comparable in studies with a low overall risk of bias (32%, 95% CI 18–46%, *I*^2^ 96%).

## 4. Discussion

### 4.1. Main Findings

We identified 23 studies evaluating the neonatal outcomes in infants born with isolated OCHDs and LBW or VLBW. The overall summary of mortality before discharge for isolated OCHDs was 37% (95%CI 27–47%). Mortality varied substantially across CHD subtypes, with a high mortality for HLHS and TAPVR followed by TGA and CoA, but the difference was not significant. Mortality was significantly higher in population-based studies compared to hospital-based studies (49% [36–63%] vs. 10% [4–15%]).

### 4.2. Interpretation

The mortality of infants with OCHDs and an LBW/VLBW appears higher compared to the reported mortality of children without an LBW/VLBW. Although no direct comparison can be made here, mortality seems two- to four-fold higher in infants with LBW/VLBW: HLHS (50% vs. 25%), TAPVR (47% vs. 12%), TGA (34% vs. 4%) and CoA (16% vs. 4%) [42].

A preliminary hypothesis could be that mortality is mainly correlated to the complexity of the surgical procedure, which would be increasingly difficult the smaller the anatomical structures. However, if we refer to the Aristotle operative complexity score [43], we would expect the highest mortality for HLHS (Aristotle 15), followed by TGA (Aristotle 10), then TAPVR (Aristotle 9), and finally, CoA (Aristotle 6); this does not align with our findings. In addition, numerous surgical successes in LBW/VLBW infants suggest that surgical technique is not the major obstacle to managing these children [24,44,45]. Additionally, the lack of an increased early re-intervention rate [34] in LBW/VLBW infants indirectly suggests that surgical outcomes were sufficient. Hence, technical surgical factors are probably not the primary cause of higher mortality in this specific LBW/VLBW population.

Mortality may also reflect challenges during post-operative management, where prematurity is reported as an independent risk factor in children with CHDs [46]. Indeed, these LBW/VLBW infants are also mostly born preterm, with organ immaturity increasing the risk of post-operative complications [31,46]. Gut immaturity and the occurrence of NEC are reported as one of the leading causes of death in preterm infants with CHDs [46]. For example, the adjusted odds ratio for the development of NEC in VLBW neonates with CHDs range from 1.3 to 1.8 [29,31], with mortality up to 60% [31,46]. Hemodynamic impairment of some CHDs associated with gut immaturity may further predispose these infants to NEC. Thus, cardiac physiologies impairing gut perfusion, i.e., CHDs with overt (e.g., HLHS) [47] or potential (e.g., CoA, TAPVR) [48] heart failure may, therefore, increase the occurrence of NEC, with a relative risk reported across cohorts of up to 3.7 [49]. The balance between gut maturity and mesenteric perfusion thus appears to be highly precarious in these children such that even slightly lower gestational age children with CHDs may be at a higher risk for NEC [50]. Acute kidney injury may affect 40 to 60% of children with CHDs requiring surgery [51,52], and it is strongly associated with increased mortality [53]. This large proportion of acute kidney injury appears to be favored by impaired renal blood flow due to heart failure in some OCHDs, prolonged bypass time, fluid overload, and inflammation [46]. Noteworthy, no study has yet assessed the potential additive effect of preterm birth [54] and CHD [51] on mortality.

The preterm infant’s cerebral and pulmonary immaturity are also major causes of death in preterm infants [51]. Hence, we could assume that CHDs with altered hemodynamics (e.g., heart failure, increased pulmonary blood flow), a need for mechanical ventilation, and high oxygen exposure [55] could lead to consequential vascular damages in preterm OCHDs. Therefore, we would expect a higher proportion of intraventricular hemorrhage (through germinal matrix vasculature damages), ischemic lesions, BPD, and pulmonary hypertension in preterm infants with OCHDs. However, currently, to our knowledge, such adverse outcomes are not reported.

Pre-operative morbidity as a factor worsening post-operative management should also be discussed. Any element that influences the pre-operative status and allows the prevention of precarious hemodynamic states is essential. Additionally, prenatal diagnosis is one of the aspects of managing these patients that could be impactful. Although the benefit of prenatal diagnosis on early mortality [56,57,58] is still debated, its impact on pre-operative morbidity and post-operative management is more consistent [52,59,60,61]. Thus, prenatal diagnosis could be decisive in children born with an LBW/VLBW with CHDs for whom the post-operative risk is increased. The difference in mortality observed among LBW/VLBW infants with OCHDs may be partly explained by the difference in neonatal screening rates for these OCHDs. Thus, despite having a higher surgical complexity score than TAPVR, TGA appears to have a lower mortality, likely explained in part by a higher screening rate (<8% for TAPVR vs. 45% for TGA) [62].

The observed difference between the hospital- and population-based studies may be attributed to selection and survival biases. Indeed, we expect higher mortality in exhaustive population-based studies (some of them even including the prenatal period) [21,26] compared with surgical series that may exclude all pre-operative deaths [9,28,34]. The measurement bias inherent to retrospective hospital-based cohorts compared to population-based prospective cohorts may also contribute to the difference in mortality. Moreover, hospital-based studies publishing their results are usually the reference centers, with major expertise in CHD diagnosis and repair; this could explain the low mortality they report. Even if the funnel plot did not provide statistical evidence of publication bias, it remains possible that some series of patients with a worse prognosis were unpublished.

### 4.3. Limitations

Our study has limitations. First, given the low prevalence of certain critical isolated OCHDs, we could not include all OCHD subtypes in our review and meta-analysis. Because of the lack of data, OCHDs with a potentially better immediate prognosis were not included. On the other hand, we found no data on the more complex CHDs requiring neonatal interventions, such as the TGA-CoA-ventricular septal defects, which may have a poorer prognosis.

Second, the generalizability of our findings to middle- and low-income countries where medical resources and surgical expertise are in short supply is limited. This is because studies included in this review were from high-resource western countries, mostly from the USA. There was a trend in mortality across CHD subtypes, but the difference was not statistically significant (*p* = 0.13). However, it is common knowledge that meta-regression analyses have low power, and we believe that the differences we observed might still reflect clinically relevant trends. In addition to this, the use of large and administrative databases in several studies could be a source of inaccuracies due to coding and/or data entry errors.

Finally, we were limited by the data available in the studies, and consequently we were unable to assess pooled mortality for preterm and SGA infants (although outcomes of SGA infants are presented in the Supplementary Materials: Table S5). Had the data been available, pooled mortality using individual patient data from the different studies may have been more informative, especially with regard to the possible impact of preterm birth and SGA (through the use of birthweight adjusted for gestational age). However, while VLBW babies are all premature, not all preterm births are of a VLBW, and further investigation into the mortality of preterm infants with CHDs is required.

## 5. Conclusions

The mortality of children born with OCHD and LBW/VLBW is high and appears substantially higher than that of children without LBW/VLBW. The impact of LBW/VLBW on the morbidity and mortality of some OCHD subgroups seems critical. The question of pre-operative morbidity and consequently the impact of prenatal diagnosis on post-operative outcomes deserves to be evaluated in this vulnerable population.

## Figures and Tables

**Table 3:** Early mortality in infants born with severe neonatally operated congenital heart defects and low or very-low birthweight: Stratified analysis with meta-regression.

Stratified Analysis	No. of Study Cohorts	Summary Mortality, % (95%CI)	*I*^2^, %	Meta-Regression	
				Odds Ratio (95% CI)	*p* Value
Specific CHD					0.13
Coarctation of the aorta	8	16 (6–25)	88	Reference	
Transposition of the great arteries	6	34 (11–58)	90	2.6 (0.5–12.8)	
Hypoplastic left heart syndrome	10	50 (30–69)	97	4.4 (1.1–17.7)	
Total anomalous pulmonary venous return	6	47 (26–72)	66	5.1 (0.9–12.8)	
**Birthweight**					0.003
Low birthweight	10	16 (6–25)	93	Reference	
Moderately low birthweight	3	15 (0–36)	68	0.77 (0.1–4.7)	
Very-low birthweight	17	56 (38–73)	95	6.0 (2.3–15.6)	
**Study design**					0.006
Hospital-based	10	10 (4–15)	17	Reference	
Population-based	20	49 (36–63)	97	4.9 (1.6–14.5)	
**Overall meta-analysis**	**30**	**37 (27–47)**	**96**	**N/A**	

Legend: Low birthweight (i.e., <2500 g); moderately low birthweight (i.e., 1500–2499 g), very-low birthweight (i.e., <1500 g).

## Data Availability

Data are contained within the article and supplementary material.

## Supplementary Materials

The following supporting information can be downloaded at: https://www.mdpi.com/article/10.3390/jcdd10020047/s1, Table S1: PRISMA 2020 checklist; Table S2: Discrepancies between the protocol (PROSPERO CRD4202170289) and the final review; Table S3: The detailed literature search strategy; Table S4: A summary of the comparable outcomes in the studies not used in the meta-analysis; Table S5: The results of the studies specifically on the adverse outcomes in infants with SGA and CHDs.

## Abbreviations

AS	Aortic stenosis
BPD	Bronchopulmonary Dysplasia
CASP	Critical Appraisal Skills Program
CHD	Congenital heart defects
CoA	Coarctation of the aorta
CI	Confidence interval
HLHS	Hypoplastic left heart syndrome
IVH	Intraventricular hemorrhage
LBW	Low birth weight
MLBW	Moderately low birth weight
NEC	Necrotizing enterocolitis
OCHD	Neonatally operated congenital heart defects
SGA	Small for gestational age
TGA	Transposition of the great arteries
TAPVR	Total anomalous pulmonary venous return
VLBW	Very-low birth weight
