# Supplementary material for: Early Mortality in Infants Born with Neonatally Operated Congenital Heart Defects and Low or Very-Low Birthweight: A Systematic Review and Meta-Analysis
Source: J Cardiovasc Dev Dis. 2023 Jan 27;10(2):47. doi: 10.3390/jcdd10020047 (PMC9966697; doi:10.3390/jcdd10020047)
Supplement: Supplementary file 1 [file jcdd-10-00047-s001.zip › jcdd-2118187-supplementary.pdf]

Supplementary materiels:
Table S1. PRISMA 2020 checklist (1/3)

Section and Topic	Item #	Checklist item	Location where item is reported
**TITLE**			
Title	1	Identify the report as a systematic review.	P1
**ABSTRACT**			
Abstract	2	See the PRISMA 2020 for Abstracts checklist.	P2
**INTRODUCTION**			
Rationale	3	Describe the rationale for the review in the context of existing knowledge.	P4
Objectives	4	Provide an explicit statement of the objective(s) or question(s) the review addresses.	P4
**METHODS**			
Eligibility criteria	5	Specify the inclusion and exclusion criteria for the review and how studies were grouped for the syntheses.	P5
Information sources	6	Specify all databases, registers, websites, organisations, reference lists and other sources searched or consulted to identify studies. Specify the date when each source was last searched or consulted.	P6;P9
Search strategy	7	Present the full search strategies for all databases, registers and websites, including any filters and limits used.	P36
Selection process	8	Specify the methods used to decide whether a study met the inclusion criteria of the review, including how many reviewers screened each record and each report retrieved, whether they worked independently, and if applicable, details of automation tools used in the process.	P6
Data collection process	9	Specify the methods used to collect data from reports, including how many reviewers collected data from each report, whether they worked independently, any processes for obtaining or confirming data from study investigators, and if applicable, details of automation tools used in the process.	P6
Data items	10a	List and define all outcomes for which data were sought. Specify whether all results that were compatible with each outcome domain in each study were sought (e.g. for all measures, time points, analyses), and if not, the methods used to decide which results to collect.	P6;P7
	10b	List and define all other variables for which data were sought (e.g. participant and intervention characteristics, funding sources). Describe any assumptions made about any missing or unclear information.	P7
Study risk of bias assessment	11	Specify the methods used to assess risk of bias in the included studies, including details of the tool(s) used, how many reviewers assessed each study and whether they worked independently, and if applicable, details of automation tools used in the process.	P7
Effect measures	12	Specify for each outcome the effect measure(s) (e.g. risk ratio, mean difference) used in the synthesis or presentation of results.	P7
Synthesis methods	13a	Describe the processes used to decide which studies were eligible for each synthesis (e.g. tabulating the study intervention characteristics and comparing against the planned groups for each synthesis (item #5)).	P7
	13b	Describe any methods required to prepare the data for presentation or synthesis, such as handling of missing summary statistics, or data conversions.	N/A
	13c	Describe any methods used to tabulate or visually display results of individual studies and syntheses.	P7

Table S1 (continued). PRISMA 2020 checklist (2/3)

Section and topic	Item #	Checklist item	Location where item is reported
Synthesis methods	13d	Describe any methods used to synthesize results and provide a rationale for the choice(s). If meta-analysis was performed, describe the model(s), method(s) to identify the presence and extent of statistical heterogeneity, and software package(s) used.	P7;P8
	13e	Describe any methods used to explore possible causes of heterogeneity among study results (e.g. subgroup analysis, meta-regression).	P7;P8
	13f	Describe any sensitivity analyses conducted to assess robustness of the synthesized results.	P8
Reporting bias assessment	14	Describe any methods used to assess risk of bias due to missing results in a synthesis (arising from reporting biases).	N/A
Certainty assessment	15	Describe any methods used to assess certainty (or confidence) in the body of evidence for an outcome.	N/A
**RESULTS**			
Study selection	16a	Describe the results of the search and selection process, from the number of records identified in the search to the number of studies included in the review, ideally using a flow diagram.	P9; P27
	16b	Cite studies that might appear to meet the inclusion criteria, but which were excluded, and explain why they were excluded.	P9; P27
Study characteristics	17	Cite each included study and present its characteristics.	P9; P23-24
Risk of bias in studies	18	Present assessments of risk of bias for each included study.	P10; P25
Results of individual studies	19	For all outcomes, present, for each study: (a) summary statistics for each group (where appropriate) and (b) an effect estimate and its precision (e.g. confidence/credible interval), ideally using structured tables or plots.	P10-11; P26; P28
Results of syntheses	20a	For each synthesis, briefly summarise the characteristics and risk of bias among contributing studies.	P10; P25
	20b	Present results of all statistical syntheses conducted. If meta-analysis was done, present for each the summary estimate and its precision (e.g. confidence/credible interval) and measures of statistical heterogeneity. If comparing groups, describe the direction of the effect.	P10-11; P26; P28
	20c	Present results of all investigations of possible causes of heterogeneity among study results.	P26; P28
	20d	Present results of all sensitivity analyses conducted to assess the robustness of the synthesized results.	P26; P28
Reporting biases	21	Present assessments of risk of bias due to missing results (arising from reporting biases) for each synthesis assessed.	N/A
Certainty of evidence	22	Present assessments of certainty (or confidence) in the body of evidence for each outcome assessed.	N/A

Table S1 (continued). PRISMA 2020 checklist (3/3)

Section and topic	Item #	Checklist item	Location of where item is reported
**DISCUSSION**			
Discussion	23a	Provide a general interpretation of the results in the context of other evidence.	P12-14
	23b	Discuss any limitations of the evidence included in the review.	P14-15
	23c	Discuss any limitations of the review processes used.	P15
	23d	Discuss implications of the results for practice, policy, and future research.	P15
**OTHER INFORMATION**			
Registration and protocol	24a	Provide registration information for the review, including register name and registration number, or state that the review was not registered.	P5
	24b	Indicate where the review protocol can be accessed, or state that a protocol was not prepared.	P5;P17
	24c	Describe and explain any amendments to information provided at registration or in the protocol.	P33
Support	25	Describe sources of financial or non-financial support for the review, and the role of the funders or sponsors in the review.	P16
Competing interests	26	Declare any competing interests of review authors.	P16
Availability of data, code and other materials	27	Report which of the following are publicly available and where they can be found: template data collection forms; data extracted from included studies; data used for all analyses; analytic code; any other materials used in the review.	N/A

Legend: P page; N/A not applicable

Table S2. Discrepancies between the protocol (PROSPERO CRD4202170289) and the final review

Discrepancy	Protocol	Final review
**Study question**	What are the adverse outcomes in growth restricted infants with CHD? How are adverse outcomes affected by the severity of growth restriction affect and type of CHD?	What are the adverse outcomes in Low birth weight and Very low birth weight infants?
**Search date**	inception up until 29/02/2020	inception up until 13/10/2021
**Condition or domain being studied**	Isolated CHD, i.e., CHD not associated with any known chromosomal, genetic or other anomalies or syndromes.	Isolated CHD and certain specific CHD with a low percentage of chromosomal, genetic or other anomalies or syndromes
**Main outcome (s)**	Mortality and a range of morbidity indicators	Main focus: mortality 1 month after surgical intervention.
**Strategy for data analysis**	Meta-analysis of pooled OR. Analysis to be carried out on Stata 12.1. Heterogenity assessed using I^2statistic and Forest plots	Meta-analysis of proportionate mortality. Analysis carried out on Stata 15. Heterogenity assessed using I^2 statistic, Forest plot and metaregression.

Table S3. Detailed literature search strategy

**Database**	**Search string**
**Pubmed**	(((((((((((((((((((((((("congenital cardiac"[Title/Abstract]) OR "congenital cardiovascular"[Title/Abstract]) OR "congenital heart anomalies"[Title/Abstract]) OR "congenital heart malformations"[Title/Abstract])) OR "Heart Septal Defects"[Title/Abstract]) OR "Truncus Arteriosus"[Title/Abstract])) OR "Common arterial trunk"[Title/Abstract]) OR "Aortic Valve Stenosis"[Title/Abstract]) OR "Transposition of Great Vessels"[Title/Abstract])) OR "Aortic Coarctation"[Title/Abstract]) OR "Hypoplastic Left Heart Syndrome"[Title/Abstract]) OR "Pulmonary Valve Stenosis"[Title/Abstract])) OR "Tetralogy of Fallot"[Title/Abstract]) OR "Atrioventricular Septal Defect"[Title/Abstract]) OR "Congenital heart defect"[Title/Abstract]) OR "Congenital heart disease"[Title/Abstract])) OR (("Heart Defects, Congenital"[Mesh]) OR "Pulmonary Atresia"[Mesh])))) AND ((((((("Fetal Growth Retardation"[Title/Abstract]) OR "Small for Gestational Age"[Title/Abstract]) OR "Low Birth Weight"[Title/Abstract]) OR "Fetal Growth Restriction"[Title/Abstract])) OR ((("Infant, Low Birth Weight"[Mesh]) OR "Infant, Small for Gestational Age"[Mesh]) OR "Fetal Growth Retardation"[Mesh]))) Filters: Humans
**Embase**	'congenital heart malformation' AND ('intrauterine growth retardation' OR 'small for date infant'/exp) AND [embase]/lim NOT ([embase]/lim AND [medline]/lim)

Table S4. Summary of comparable outcomes in studies on LBW and VLBW not used in the meta-analysis

Outcome	Author	Year	Term (weeks)	Object	CHD	Result	95% CI	Measurement
**mortality**								
90 day postop	Curzon	2008	all	LBW	HLHS	1.4	1.0-2.0	Risk Ratio
					TAPVR	3.0	1.4-6.2	
					TGA	4.6	1.0-22.0	
					CoA	2.65	1.1-6.2	
**Survival**								
5 year	Bacha	2001	PT (26-35)	VLBW	CoA	80%	70%-90%*	Kaplan meier actuarial estimate
5 year	Best	2017	term	LBW	all i.CHD	95.2%	94.2%-96.3%	conditional survival estimate
			very PT			78.8%	72.8%-84.7%	
			term	LBW	Severe CHD type1 (includes HLHS)	53.5%	33.0%-73.9%	
			PT	LBW		32.5%	7.4% - 57.5%	
			term	LBW	ToF	89.3%	82.2%-96.4%	
			PT	LBW		84.4%	73.0%-96.0%	
			term	LBW	TGA	92.5%	86.4 -98.6%	
			PT	LBW		72.5%	51.1%-93.9%	
			term	LBW	CoA	90.2%	82.4%-98.0%	
			PT	LBW		79.9%	62.0%-97.8%	
1 year post op	Hirsch	2011	PT	LBW	HLHS	55%		
1 year	Karamlou	2009	all	LBW	CoA	76% overall (67% before 1999 and 90% after)	not stated	Kaplan meier survival curve
6 months	Manchego	2018	PT	LBW	HLHS	40%	not stated	proportions
					CoA	87%	not stated	proportions
					TGA	76%	not stated	

Table S4 (continued). Summary of comparable outcomes in studies not used in the meta-analysis (2/3)

Outcome	Author	Year	Term	Object	CHD	Result	95% CI	Measurement
**Survival**								
**6 year**	Miller	2019	all	LBW	HLHS	1.7	1.2-2.4	Hazards ratio
Midterm	Murphy	2015	all	LBW	HLHS	53%	not stated	proportion
1 year	Oster	2013	all	LBW	CCHD	1.74	1.34-2.24	Hazards ratio
Overall survival overtime between 1979-2005	Siffle	2015	all	VLBW	HLHS	0%	/	proportion
				LBW		15.6%	5.7%-30.0%	
**Necrotizing enterocolitis**								
	Bain	2014	PT	VLBW	ASD	1.26	1.06-1.49	adjusted Odds Ratio
					VSD	1.27	1.08-1.52	
					ASD and VSD	1.80	1.03-3.12	
	Pappas	2012	PT	VLBW	all CHD[+]	1.23	0.73-2.04	adjusted Risk Ratio
**Neurodevelopment**								
Bayley mental development index evaluated at 18-22 months	Pappas	2012	PT	VLBW	all CHD[+]	score <70: 1.61	1.21-2.13	adjusted Risk Ratio
Psychomotor development index						score <70:1.44	0.93-2.21	
Neurodevelopment impairement						score < 70: 1.45	1.13-1.87	
neurological disease	Roussin	2007	PT	VLBW	TGA	n=1	/	proportion

Table S4 (continued). Summary of comparable outcomes in studies not used in the meta-analysis (3/3)

Outcome	Author	Year	Term	Object	CHD	Result	95% CI	Measurement
**Neurodevelopment**								
Evaluated at 6 years using BASC adaptive skill composite score	Miller	2019	all	LBW	HLHS	female: 40<br>male: 50	21-55<br>35-62	mean standard deviation
Vineland II score:				LBW		adaptive behaviour composite: 90	70-115	
						communication: 85	75-125	
						daily living skills: 90	60-120	
						motor skills: 80	60-110	
						socialization: 100	75-130	
**Length of mechanical ventilation**								
	Kalfa	2015	all	LBW	HLHS	7.5	1-35	median days
	Kalfa	2014	all		all CHD[+]	4	1-32	days
Prolonged ventilation	Roussin	2007	PT	VLBW	TGA	n=8	/	proportion

Table S5. Results of studies on specifically on the adverse outcomes in infants with SGA and CHD (1/2)

Outcome	Author	Year	Term (weeks)	CHD	Result	95% CI	Measurement
**Mortality**							
During hospitalisation	El Hassan	2018	all	HLHS	27.6%		Proportion
6 year mortality after Norwood procedure	Miller	2019	all	HLHS	0.93	0.86-1.02	Hazards ratio
Hospital mortality after surgery	Roussin	2007	PT	TGA	30.7%		Proportion
1 year	Steurer	2018	PT (<32)	CCHD	1.6	0.8-3.4	adjusted Odds Ratio
			PT (32-33)		2.3	0.9-6.2	
			PT (34)		0.9	0.3-3.0	
			PT (35)		1.8	0.8-4.4	
			PT (36)		1.7	0.9-3.3	
			term (37)		2.8	1.7-4.5	
			term (38)		1.6	1.1-2.6	
			term (39)		1.9	1.3-2.8	
			term (40)		2.6	1.5-4.4	
			term (41)		1.3	0.4-4.4	
**Survival**							
Transplant free survival from birth through Fontan palliation	Gelehrter	2011	PT	HLHS	18%	not stated	proportion
Live at discharge	Story	2015	all	all i.CHD	82%	not stated	proportion
**Necrotizing enterocolitis**							
	El Hassan	2018	all	HLHS	10.6%	Not stated	proportion
	Story	2015	all	all i.CHD	6%	not stated	proportion

Table S5 (continued). Results of studies specifically on the adverse outcomes in infants with SGA and CHD (2/2)

Outcome	Author	Year	Term	CHD	Result	95% CI	Measurement
**Neurodevelopment**							
Cognitive impairment: KABC global score ≥ 1 SD below normative value evaluated at 3 years	Calderon	2017	all	all i.CHD	no surgery group : 1.3 cardiac surgery group:5.9	0.5-3.6 1.7-20.1	adjusted Odds Ratio
Neurodevelopment evaluated at 6 years using BASC adaptive skill composite score	Miller	2019	all	HLHS	female and male 45	38-58	mean standard deviation
Neurodevelopment evaluated at 6 years Vineland scores					adaptive behaviour composite: 90	80-120	
					communication: 100	82-122	
					daily living skills: 90	80-120	
					motor skills: 85	75-100	
					socialization: 100	80-125	

Legend: ; § population based study; + preterm births only; i.CHD isolated CHD; CCHD critical CHD; LBW low birthweight; N CHD total number of congenital heart defects; NEC necrotizing enterocolitis; SGA small for gestational age;VLBW very low birthweight; HLHS hypoplastic left heart syndrome;a; TGA transposition of the great arteries
